# RAF and MEK Inhibitors in Non-Small Cell Lung Cancer

**DOI:** 10.3390/ijms25094633

**Published:** 2024-04-24

**Authors:** Christos Adamopoulos, Kostas A. Papavassiliou, Poulikos I. Poulikakos, Athanasios G. Papavassiliou

**Affiliations:** 1 Department of Biological Chemistry, Medical School, National and Kapodistrian University of Athens, 11527 Athens, Greece; 2 Department of Oncological Sciences, Precision Immunology Institute, Tisch Cancer Institute, Icahn School of Medicine at Mount Sinai, New York, NY 10029, USA; poulikos.poulikakos@mssm.edu; 3 First University Department of Respiratory Medicine, ‘Sotiria’ Hospital, Medical School, National and Kapodistrian University of Athens, 11527 Athens, Greece; konpapav@med.uoa.gr

**Keywords:** lung cancer, non-small cell lung cancer, BRAF inhibitors, MEK inhibitors, BRAFV600E, targeted therapies, MAPK pathway

## Abstract

Lung cancer, despite recent advancements in survival rates, represents a significant global health burden. Non-small cell lung cancer (NSCLC), the most prevalent type, is driven largely by activating mutations in Kirsten rat sarcoma viral oncogene homologue (KRAS) and receptor tyrosine kinases (RTKs), and less in v-RAF murine sarcoma viral oncogene homolog B (BRAF) and mitogen-activated protein-kinase kinase (MEK), all key components of the RTK-RAS-mitogen-activated protein kinase (MAPK) pathway. Learning from melanoma, the identification of *BRAFV600E* substitution in NSCLC provided the rationale for the investigation of RAF and MEK inhibition as a therapeutic strategy. The regulatory approval of two RAF-MEK inhibitor combinations, dabrafenib–trametinib, in 2017, and encorafenib–binimetinib, in 2023, signifies a breakthrough for the management of BRAFV600E-mutant NSCLC patients. However, the almost universal emergence of acquired resistance limits their clinical benefit. New RAF and MEK inhibitors, with distinct biochemical characteristics, are in preclinical and clinical development. In this review, we aim to provide valuable insights into the current state of RAF and MEK inhibition in the management of NSCLC, fostering a deeper understanding of the potential impact on patient outcomes.

## 1. Introduction

Lung cancer remains the leading cause of cancer-related mortality worldwide, accounting for 18% of all cancer-related deaths, despite the increased survival rates over the last years [1,2]. In the United States alone, it will account for an estimated 125,070 deaths in the year 2024 [3]. Lung cancer is broadly divided histologically into non-small cell lung cancer (NSCLC), which accounts for 85% of the cases, and small cell lung cancer (SCLC). NSCLC is categorized further into several histological subtypes, of which the most prevalent are lung adenocarcinoma (LUAD), lung squamous cell carcinoma (LSCC), and large cell carcinoma accounting for about 40%, 25%, and 10% of NSCLC cases, respectively [4,5]. The complex molecular landscape in NSCLC comprises genetic alterations in a wide range of genes coding mainly for key and druggable components of the receptor tyrosine kinase (RTK)-RAS-mitogen-activated protein kinase (MAPK) axis. These include Kirsten rat sarcoma (KRAS), epidermal growth factor receptor (EGFR), v-RAF murine sarcoma viral oncogene homolog B (BRAF), anaplastic lymphoma kinase (ALK), c-ROS oncogene-1 (ROS1), hepatocyte growth factor receptor (HGFR or MET), human epidermal growth factor receptor 2 (HER2), rearranged during transfection (RET), and neurotrophic tropomyosin receptor kinase (NTRK) [6,7]. All of these molecular aberrations can drive lung cancer oncogenesis. The implementation of targeted therapies in the clinical practice based on molecular profiling of NSCLC patients has led to a significant improvement in lung cancer survival rates [1,8,9]. The identification of BRAF mutations, particularly the substitution V600E in about 4% of NSCLC, and the clinical success of BRAF and MEK inhibitors in the context of metastatic *BRAFV600E*-mutant melanoma set the rationale for the clinical investigation of these drugs as a therapeutic strategy targeting *BRAFV600E*-mutant NSCLC. Over the last years, the Food and Drug Administration (FDA) has granted approval for BRAF and MEK inhibitor combinations for the treatment of *BRAFV600E*-mutant NSCLC. These include the 2017 approval for the combination of dabrafenib (BRAF inhibitor) with trametinib (MEK inhibitor) and the more recent (2023) approval for the combination of encorafenib (BRAF inhibitor) with binimetinib (MEK inhibitor). Despite these advancements, notable challenges persist whenn utilizing BRAF and MEK inhibitors for the management of *BRAF* mutant-driven lung cancer. Foremost among these challenges is the emergence of adaptive resistance to BRAF and MEK inhibition, which hinders therapeutic efforts [10,11]. Secondarily, the use of these inhibitors is limited to the case of *BRAFV600E*-mutant NSCLC, which has a low frequency among lung cancer cases. There is an urgent need for the development of rationally designed combinatorial treatment strategies that can overcome the development of drug resistance, while simultaneously expanding therapeutic indications, to *BRAF non-V600-*, *KRAS-*, or *RTK*-mutant NSCLC. The next generation of RAF and MEK inhibitors, with distinct biochemical properties, are already under preclinical and clinical evaluation for the treatment of lung cancer. This review aims to explore the current state of knowledge and therapeutic advancements of RAF and MEK inhibitors’ evaluation in preclinical and clinical cases of mostly *BRAF*-mutant lung cancer hoping to provide new insights for a more precise and rational therapeutic design and pharmacological intervention.

## 2. The RTK/RAS/MAPK Pathway in Lung Cancer

### 2.1. RTK/RAS/MAPK Pathway

**Figure 1:**
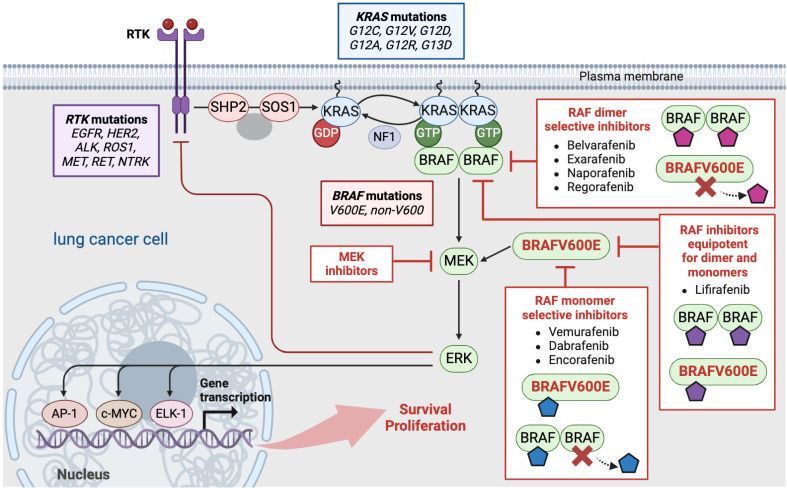
The RTK/RAS/MAPK pathway, its key components and mutations in lung cancer and the different categories of RAF inhibitors based on their distinct structural properties. RTK, receptor tyrosine kinase; SHP2, Src homology 2 domain-containing phosphatase 2; SOS1, son of sevenless homolog; KRAS, Kirsten rat sarcoma viral oncogene homolog; GDP, guanosine diphosphate; GTP, guanosine triphosphate; BRAF, v-RAF murine sarcoma viral oncogene homolog B; MEK, mitogen-activated protein-kinase kinase; ERK, extracellular signal-regulated kinase; AP-1, activator protein-1; c-MYC, cellular Myelocytomatosis oncogene; ELK-1, ETS like-1; NF1, neurofibromin 1; *EGFR*, epidermal growth factor receptor; *ALK*, anaplastic lymphoma kinase; *ROS1*, c-ros oncogene-1; *MET*, hepatocyte growth factor receptor; *HER2*, human epidermal growth factor receptor 2; *RET*, rearranged during transfection; *NTRK*, neurotrophic tropomyosin receptor kinase. This figure was created using the tools provided by BioRender.com (accessed on 10 April 2024).

The RTK/RAS/MAPK pathway is a fundamental signal transduction cascade which, in normal cells, conveys extracellular signals from cell surface receptors, mainly RTKs, intracellularly to the nucleus to promote several cellular functions such as survival, cell growth, and differentiation [12,13]. Under normal conditions, stimulation of RTKs, usually upon growth factor binding, results in their intracellular activation and in the recruitment and activation of adaptor proteins such as the Src homology region 2 domain-containing phosphatase 2 (SHP2) and the growth factor receptor-bound protein 2 (GRB2). These adaptor proteins transmit signals that result in the recruitment and activation of guanine nucleotide-exchange factors (GEFs), such as son of sevenless homologue 1 (SOS1). GEFs directly activate the membrane-bound small GTPase RAS, switching it from an inactive GDP-bound form to an active GTP-bound form. Active RAS in turn recruits and activates, through dimerization and phosphorylation, members of the rapidly accelerated fibrosarcoma (RAF) kinase family (ARAF, BRAF, and CRAF) through interaction with their N-terminal RAS-binding domain. The dimerization of two RAF protomers results in the formation of a catalytically active protein dimer [12,14]. The formed catalytically active RAF dimer is stabilized structurally through an inward movement of the αC-helix [15,16]. Activated RAF facilitates phosphorylation and activation of MEK1/2, which can then phosphorylate and activate ERK1/Activated ERK1/2, and can then phosphorylate several downstream targets of the MAPK pathway, such as transcriptions factors, including AP-1, c-MYC, and ELK-1, that control cell survival and proliferation [17,18,19]. At the same time, activated ERK induce multiple negative feedback mechanisms to regulate excessive MAPK pathway activity. These include, among other mechanisms, the immediate suppression of RTKs’ expression upstream of RAF [11,20]. Oncogenic mutations in key components of the RTK/RAS/MAPK pathway drive up to 30% of all human cancers. NSCLC, specifically, is driven by, usually mutually exclusive, mutations in RTKs, such as *EGFR* (1–15%, in non-Asian populations), HER2 (2–4%), *ALK* (3–7%), ROS1 (1–4%), KRAS (25%), and BRAF (3–5%) (Figure 1) [6,9,18,21,22,23].

### 2.2. BRAF Inhibitors

The discovery of BRAF mutations, particularly *BRAFV600E* mutation, as drivers in various cancers [24] provided a rationale for pharmacological targeting of the BRAF oncoprotein. Therefore, RAF inhibitors were developed aiming to disrupt the aberrant oncogenic signaling arising from mutated BRAF. The first-generation, ATP-competitive RAF inhibitors showed disappointing clinical results, mainly because of poor MAPK pathway inhibition and poor target selectivity [11,25,26]. Next, second-generation, ATP-competitive RAF inhibitors were developed, which have increased selectivity for RAF. These RAF inhibitors, belonging structurally to the Type 1.5 class (αC-helix-OUT, DGF-IN) [27], include vemurafenib, dabrafenib, and encorafenib and have demonstrated impressive clinical activity [28,29] (Figure 1). Notably, this class of RAF inhibitors was effective in suppressing the MAPK pathway only in BRAFV600E-mutant cancer cells and not in BRAF-wild type cells [28,29,30]. This characteristic is responsible for their broad therapeutic window when targeting BRAFV600E tumors. Moreover, inhibitors of this class induce paradoxical activation of MAPK signaling in both BRAF-wild type normal and cancer cells [31,32,33]. The base of this paradox is the differential selectivity of these inhibitors for the monomeric versus dimeric form of BRAF. Thus, type 1.5 RAF inhibitors, herein RAF monomer-selective inhibitors, selectively bind to and inhibit RAF monomers but not dimers. The structural base of this selectivity is that upon binding, the inhibitor stabilizes RAF in a closed inactive conformation, with an outward position of the αC helix (αC-helix OUT RAF inhibitors) [15]. In the case of RAF dimers in cells, these inhibitors bind to one protomer of the dimer while transactivating the unbound protomer (negative allostery) [15]. These discoveries further uncovered the fact that the BRAFV600E oncoprotein signals as a monomer independently of upstream RTK/RAS activation and at the same time sets the base for the classification of BRAF mutations according to their RAF dimerization ability and their dependence on upstream RAS activity [11,13,16,34]. Next-generation equipotent RAF inhibitors, with equal potency for both RAF monomers and dimers, and dimer selective RAF inhibitors, with increased potency for RAF dimers, have been developed [15,35,36,37,38,39,40] (Figure 1). These inhibitors bind to both protomers of the RAF dimer, stabilizing the αC-helix in the IN position (αC-helix IN/DFG-OUT RAF inhibitors or type 2) [15], and are currently under preclinical and clinical evaluation in solid *BRAF*-mutant cancers, including lung cancer (Table 1).

### 2.3. Classification of BRAF Mutations

Class I *BRAF* mutations exclusively include the *BRAFV600* substitutions that produce constitutively activated BRAF monomers [34,41]. Class II *BRAF* mutations form constitutively active RAF dimers with intermediate to high kinase activity that signal independently of RAS, including certain non-V600 missense mutations, splice variants, in-frame deletions, and BRAF fusions [34,41]. Class III *BRAF* mutations are low-activity, kinase-impaired or kinase-dead mutants that form RAF heterodimers with wild-type CRAF and depend on RAS activity. Often, class III BRAF mutants in tumors co-exist with RAS mutations or *neurofibromin 1* (*NF1*) deletions (melanomas) or RTK upregulation (lung and colorectal cancers), highlighting the need for concurrent mechanisms for sustaining RAS activation despite ERK-dependent feedback [34].

### 2.4. BRAF Alterations in NSCLC

*BRAFV600E* mutations are found in about 2% of *BRAF*-mutant NSCLC patients [7,42,43]. *BRAFV600E* mutations in NSCLC tend to be more prevalent in cases characterized by micropapillary patterns and among female individuals with a history of smoking. Conversely, *BRAF non-V600E* mutations are more frequently associated with mucinous patterns and male individuals with a smoking history [42]. A retrospective analysis of *BRAF*-*mutant* NSCLC patients demonstrated an association of *BRAF* mutations, more significantly class II and III compared to class I, with brain metastasis in 29% of patients [44]. In a molecular characterization study of *BRAF*-mutant NSCLC, the most common *BRAF* mutations were missense mutations (90%), with half of them being variants of unknown significance [43]. Class I mutations were exclusively *BRAFV600E*, while *G469A* and *K601E* and *G466V* and *N581S* were the most common class II and III *BRAF* mutations, respectively [43]. The same analysis revealed that the most common concurrent mutations were *TP53*, *EGFR*, *KRAS*, and *NF1* mutations and that all three classes of *BRAF* mutations co-existed with the *EGFRL858R* activating mutation or *EGFR* exon 19 deletions in 10% of samples [43]. When the association between *KRAS* and *BRAF* mutations was examined, it was observed that class III *BRAF* mutations are more likely to co-occur with *KRAS* mutations compared to class I and II mutations [43]. Moreover, in another study, *BRAF*-mutant NSCLC was characterized by elevated levels of PD-L1 expression, in a reported 42% of *BRAFV600E* and 50% of non-*V600E* mutations [45]. Conversely, BRAF fusions were rarely detected in 0.2% of a total of 17,128 NSCLC samples, with the most prevalent fusion partners including acylglycerol kinase (AGK), dedicator of cytokinesis protein 4 (DOCK4), and tripartite motif-containing 24 (TRIM24), while the most frequently co-occurring mutations were *TP53*, *CDKN2A*, *EGFR*, and *CDKN2B* [46]. The prognostic value of *BRAFV600E* in NSCLC, given its low frequency, is still unclear. However, it has been associated with patients with poor outcomes and low response rates to platinum-based chemotherapy [47].

### 2.5. MEK Inhibitors

As of now, the FDA has granted approval to four MEK inhibitors: trametinib, selumetinib, cobimetinib, and binimetinib. These small molecule inhibitors are allosteric non-competitive inhibitors of MEK and were the first selective inhibitors of the MAPK pathway to enter the clinic [48]. Trametinib, as a single agent, was approved in 2013 by the FDA for the treatment of metastatic *BRAFV600E/K*-mutant melanoma patients who have not been previously treated with BRAF inhibitors [49]. Additionally, trametinib is approved in combination with dabrafenib for metastatic patients with *BRAFV600E/K*-mutant melanoma and *BRAFV600E*-mutant NSCLC [50], and pediatric patients with *BRAFV600E*-mutant low-grade glioma with a *BRAFV600E* mutation. Selumetinib was granted approval for pediatric patients (2 years old and older) with *neurofibromatosis type 1 (NF1)* who present symptomatic, inoperable plexiform neurofibromas [51]. Cobimetinib is approved in combination with vemurafenib for unresectable or metastatic *BRAFV600E/K*-mutant melanoma or as a single agent for histiocytic neoplasms [52], while binimetinib in combination with encorafenib is approved for unresectable or metastatic *BRAFV600E/K*-mutant melanoma and metastatic *BRAFV600E*-mutant NSCLC [53]. Biochemically, allosteric MEK inhibitors can be divided into two main groups. The first includes compounds that their binding to MEK promotes, with different potencies, the disruption of the RAF−MEK complex, such as trametinib and selumetinib, and a second group which comprises inhibitors that foster MEK−RAF complex formation “RAF/MEK clamps” while preventing phosphorylation by RAF, such as avutometinib [40,54,55,56]. Moreover, although the majority of MEK inhibitors bind to MEK in a similar fashion, occupying the same allosteric pocket, the increased binding affinity of certain MEK inhibitors is the basis for their higher inhibitory potency [40].

### 2.6. Classification of MEK Mutations

MEK mutations are classified into three distinct types: RAF-independent, RAF-regulated, and RAF-dependent. RAF-independent MEK mutations are typically marked by in-frame deletions resulting in MEK hyperactivation. Conversely, RAF-regulated and RAF-dependent MEK mutations require RAF phosphorylation for their optimal activity [41,57].

### 2.7. MEK Alterations in NSCLC

The landscape of MEK alterations in lung cancer has remained relatively unexplored due to their extremely rare prevalence. However, when they are present in NSCLC, they are mutually exclusive with other genetic alterations [58,59]. The first study which reported MEK alterations in 2 out of 207 NSCLC patients, identified *MAP2K1-K57N* mutations and demonstrated their gain-of-function properties in vitro [58]. This was confirmed in a study which reported a 0.6% occurrence of *MEK* mutations among 6024 lung adenocarcinoma cases, revealed their mutual exclusivity with other oncogenic drivers, and demonstrated an apparent association with current smoking status [59].

## 3. BRAF and MEK Inhibitors in Lung Cancer

### 3.1. BRAF Inhibitors as Monotherapy in NSCLC

**Figure 2:**
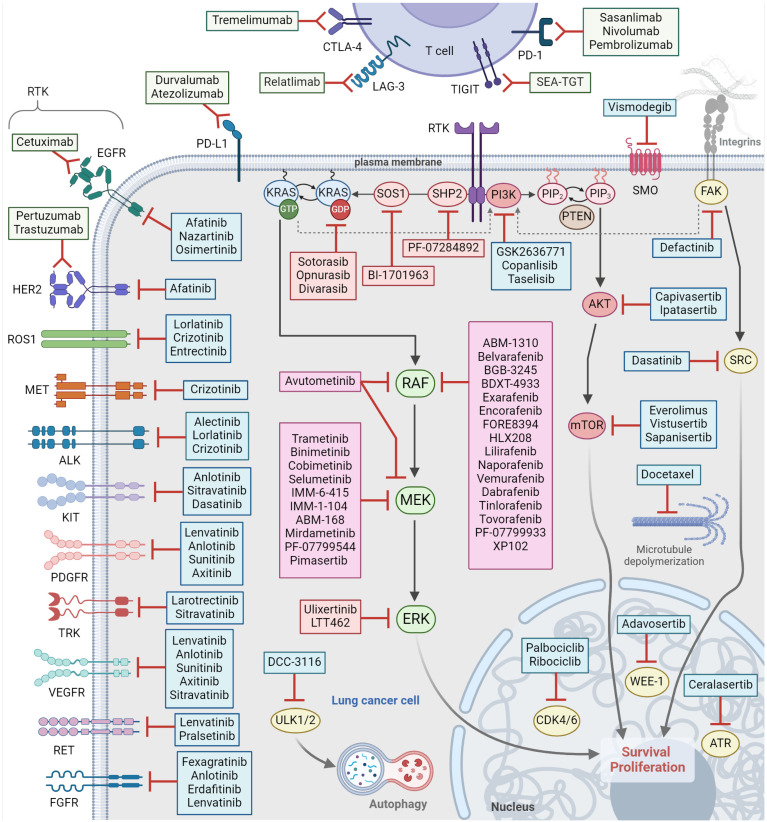
BRAF and MEK inhibitors under clinical evaluation in combination, or not, with various targeted therapies for lung cancer. RTK, receptor tyrosine kinase; SHP2, Src homology 2 domain-containing phosphatase 2; SOS1, son of sevenless homolog; KRAS, Kirsten rat sarcoma viral oncogene homolog; GDP, guanosine diphosphate; GTP, guanosine triphosphate; RAF, rapidly accelerated fibrosarcoma; MEK, mitogen-activated protein-kinase kinase; ERK, extracellular signal-regulated kinase; PI3K, phosphatidylinositol 3-kinase; PIP2/3, phosphatidylinositol 2/3; PTEN, phosphatase and tensin homolog; AKT, protein kinase B; mTOR, mechanistic target of rapamycin; EGFR, epidermal growth factor receptor; HER2, human epidermal growth factor receptor 2; ROS1, c-ros oncogene-1; MET, hepatocyte growth factor receptor; ALK, anaplastic lymphoma kinase; KIT, tyrosine-protein kinase kit; PDGFR, platelet-derived growth factor receptor; TRK, tropomyosin receptor kinase; VEGFR, vascular endothelial growth factor receptor; RET, rearranged during transfection; FGFR, fibroblast growth factor receptor; SMO, smoothened; PD-L1, programmed death-ligand 1; PD-1, programmed cell death protein 1; CTLA-4, cytotoxic T-lymphocyte-associated protein 4; LAG-3, lymphocyte-activation gene 3; TIGIT, T cell immunoreceptor with Ig and ITIM domains; FAK, focal adhesion kinase; SRC, proto-oncogene tyrosine-protein kinase; ULK1/2, unc-51-like kinase 1/2; CDK4/6, cyclin-dependent kinase 4/6; WEE-1, Wee1-like protein kinase; ATR, ataxia telangiectasia and Rad3-related protein. This figure was created using the tools provided by BioRender.com (accessed on 10 April 2024).

Since the development of the first RAF monomer-selective inhibitor, vemurafenib, and its successful implementation for the treatment of metastatic *BRAFV600E*-melanoma, these inhibitors have been evaluated in other tumor types bearing the same mutation [29,60,61]. In the context of *BRAFV600E*-mutant NSCLC, initial case reports showed a favorable response after vemurafenib administration [62,63]. The effect of vemurafenib, mainly, but also dabrafenib, was evaluated in a retrospective multicenter study in advanced *BRAF*-mutant NSCLC patients. Vemurafenib treatment showed improved outcomes, with a 54% response rate and 96% disease control, in *BRAFV600E* patients, while *non-V600E* patients had poorer outcomes [64]. These results confirm the activity of RAF monomer-selective inhibitors in patients with *BRAF*-mutant lung cancers. In a phase 2 basket trial, vemurafenib showed promise in *BRAFV600*-mutation-positive non-melanoma cancers, including NSCLC. Among NSCLC patients, 42% achieved an objective response, with a 23% progression-free survival (PFS) at 12 months. In an expanded cohort, previously untreated patients had a 37.5% objective response rate (ORR), while that of pretreated patients was 37.0%. The median duration of response (DOR) was 7.2 months. Overall, median PFS and median overall survival (OS) were 6.5 and 15.4 months, respectively [65]. Moreover, during the evaluation of vemurafenib in a phase II clinical trial, involving NSCLC patients with both *BRAFV600* and *non-V600* mutations, no objective responses were observed in the non-*V600* cohort, indicating a lack of vemurafenib activity in tumors expressing these BRAF alterations. In stark contrast, the *BRAFV600E*-cohort demonstrated a remarkable ORR of 44.8%, a 6.4-month median DOR and a 10-month overall survival [66]. Furthermore, there is some retrospective clinical evidence for vemurafenib activity in metastatic lung cancer cases, including brain and leptomeningeal metastases [67,68]. Regarding dabrafenib activity in *BRAFV600E*-mutant NSCLC, a favorable response was reported in a patient who had been enrolled in a trial for *BRAF*-mutant melanoma patients [69]. An open-label, multicenter phase II trial evaluating dabrafenib in patients with advanced *BRAFV600E*-mutant NSCLC showed improved outcomes for both treatment-naïve patients and those chemotherapy pretreated [47]. Additionally, a novel RAF dimer selective inhibitor, lifirafenib (BGB-283), tested in a phase I clinical study, demonstrated promising antitumor activity in *KRASG12*-mutant NSCLC patients [70]. Despite the evidence, although limited in some cases, of the clinical success of BRAF inhibitor monotherapy in *BRAFV600E*-mutant NSCLC, most patients developed adaptive resistance, resulting in reactivation of the MAPK pathway [71,72,73,74]. Several next-generation αC-helix IN RAF inhibitors, equipotent and dimer selective, including BGB-3245, BDTX-4933, exarafenib, and FORE8394, are currently under clinical evaluation as monotherapies for solid tumors bearing MAPK pathway mutations, including mutated NSCLC (Table 1, Figure 2) [75,76,77,78].

### 3.2. MEK Inhibitors as Monotherapy in NSCLC

Several clinical trials have investigated the role of MEK inhibitors in early clinical development for the treatment of advanced NSCLC. A phase II study compared the efficacy and safety of the MEK inhibitor AZD6244 versus the chemotherapy pemetrexed, and reported 5% and 4.5% ORRs for the MEK inhibitor-treated and chemotherapy-treated cohorts, respectively, but not significant difference in the median PFS [79]. In another phase II trial with the MEK inhibitor mirdametinib (PD-0325901), two intermittent administration schedules were tested but no objective responses were observed, and the median PFS was 1.8 months and the OS was 7.8 months [80]. Another phase II clinical trial comparing the MEK inhibitor, trametinib, to docetaxel, a microtubule depolymerization inhibitor in *KRAS*-mutant NSCLC patients, revealed no significant difference in survival outcomes [79]. However, early data from the “National Lung Matrix Trial”, testing among other targeted therapies selumetinib plus docetaxel, reported a confirmed OR for LUAD patients with *NF1* loss [81]. A phase II basket trial tested selumetinib in molecularly profiled NSCLC patients harboring mostly *KRAS* and fewer *BRAF*, *NRAS*, and *HRAS* mutations. However, only one *KRAS*-mutant patient achieved a partial response with the other nine patients failing to meet the study’s primary endpoint [82]. In general, according to these clinical trials, monotherapy with MEK inhibitors seems to provide only modest survival benefits. However, there are newer compounds, ABM-168 and IMM-6-415, with increased potency for MEK, under preclinical and clinical testing, as monotherapies for MAPK-driven cancers, including lung cancer (Table 1) [83,84].

### 3.3. BRAF and MEK Inhibitor Combinations in NSCLC

#### 3.3.1. BRAF-Mutant NSCLC

Following the clinical success from the combined use of BRAF and MEK inhibitors in *BRAFV600E*-mutant melanoma [52,85], the BRAF and MEK inhibitor combination was systematically evaluated in the context of *BRAFV600E*-mutant NSCLC. The favorable survival outcomes and safety profile of the combined dabrafenib plus trametinib treatment for *BRAFV600E*-mutant NSCLC led, in June 2017, to the FDA approval of the combination as both first and second lines, regardless of previous therapy. The approval was based on the results of a phase II clinical trial that evaluated the dabrafenib and trametinib combination in two cohorts: one treatment naïve and one with at least one prior therapy. Dabrafenib administration was evaluated as a single agent in a third cohort with at least one prior therapy [86,87]. All three cohorts exhibited favorable outcomes with the treatment-naive group showing the best responses, with an ORR of 64%, a 10.4-month DOR, and a median progression-free survival of 10.9 months [86]. Despite these positive outcomes, the combination of dabrafenib with trametinib is associated with an increased incidence of adverse effects [87]. Encorafenib is a more recently developed small molecule RAF monomer-selective inhibitor that exhibits an increased pharmacodynamic activity compared to vemurafenib or dabrafenib [88]. In 2018, the FDA granted approval for the combination of encorafenib with the MEK inhibitor binimetinib for the treatment of unresectable or metastatic melanoma patients bearing BRAF V600E/K mutations. This approval was based on the outcomes of the COLUMBUS trial (NCT01909453), a phase III randomized study involving 577 *BRAF*-mutant patients [53]. Patients who received the combination of encorafenib and binimetinib achieved a prolonged PFS compared to those treated with vemurafenib or encorafenib as monotherapy. Following that, positive results from the BEACON trial (NCT05456880) led to the FDA’s 2020 approval of the combination of encorafenib with the chimeric monoclonal anti-EGFR antibody cetuximab for the treatment of *BRAFV600E*-mutant patients with metastatic colorectal cancer [89]. Accordingly, the combination of encorafenib and binimetinib in both treatment-naive and previously treated *BRAF*-mutated NSCLC is currently being evaluated in several phase II trials (Table 1). In October 2023, the FDA granted approval to the combination of encorafenib with binimetinib for adult *BRAFV600E*-mutant NSCLC patients [90,91]. The efficacy of the combo was evaluated by the PHAROS clinical trial (NCT03915951, Table 1), an open-label, multicenter, single-arm study that enrolled 98 naïve-treated patients with metastatic *BRAFV600E*–mutated NSCLC. Preliminary findings from the PHAROS trial revealed an observed ORR of 75% in treatment-naive individuals and 46% in previously treated patients [90,91]. Another ongoing phase II clinical study (ENCO-BRAF trial; NCT04526782, Table 1) is evaluating the efficacy of the encorafenib–binimetinib combination in advanced *BRAFV600E*-mutant NSCLC patients. Furthermore, another phase II trial, testing the same combo, is enrolling metastatic NSCLC patients harboring *BRAFV600E/K/D* mutations (NCT03915951, Table 1). The primary endpoint of both studies is the ORR. The umbrella clinical trial, Landscape 1011 (NCT04585815, Table 2), is underway to assess the efficacy of sasanlimab, a novel anti-PD-1 monoclonal antibody administered subcutaneously, in combination with different targeted therapies in metastatic NSCLC patients. The first arm of the study includes *BRAFV600E*-mutant NSCLC patients who receive sasanlimab together with encorafenib plus binimetinib, with a durable ORR being the primary endpoint [92,93]. Key clinical trial outcomes for BRAF-mutant NSCLC patients upon treatment with RAF and/or MEK inhibitors, together with the most frequent adverse events, are presented in Table 3.

#### 3.3.2. KRAS-Mutant NSCLC

Recently, the FDA has granted fast track designation to the RAF-MEK clamp inhibitor avutometinib plus the KRASG12C selective inhibitor sotorasib as a treatment for KRASG12C-mutant patients previously treated with one prior line of therapy excluding treatment with a KRASG12C inhibitor [95]. The combination of avutometinib plus sotorasib is being further evaluated in the phase I/II RAMP 203 study (NCT05074810, Table 1) [96]. Additionally, the combination of avutometinib with the mTOR inhibitor everolimus in KRAS-mutant NSCLC patients has shown promising results and is being tested in another clinical trial (NCT02407509, Table 1) [97].

#### 3.3.3. EGFR-Mutant NSCLC

The MEK inhibitor selumetinib in combination with the third-generation EGFR tyrosine kinase inhibitor osmertinib has been evaluated, in parallel with the combinations of osimertinib plus savolitinib (MET inhibitor) or osimertinib plus durvalumab (anti-PD-L1 antibody), in a clinical trial investigating strategies to overcome EGFR inhibitor resistance (NCT02143466), in the context of EFGR-mutant NSCLC [98]. The ORRs were 42% for the selumetinib–osimertinib cohort, and 44% and 43% for the cohorts of osimertinib–savolitinib and osimertinib–durvalumab, respectively, but the study was discontinued due to increased reporting of interstitial lung disease in the osimertinib–durvalumab group [98]. An intermittent dosing of selumetinib in combination with osimertinib is currently under evaluation in a first-line phase II clinical study for advanced EFGR-mutant NSCLC patients (NCT03392246, Table 1).

## 4. BRAF and MEK Inhibitor-Mediated Resistance

Despite the clinical success of BRAF and MEK inhibitors, the development of adaptive and acquired drug resistance limits the effectiveness of the targeted therapy. Relief of negative feedback upon treatment with BRAF or MEK inhibitors is a major cause of adaptive drug resistance, as it results in RTK upregulation, RAS activation, and RAF dimerization promoting recovery of downstream ERK activity [10,57,99,100]. CRAF and ARAF paralogs, the other two RAF protein family members, apart from BRAF, have been identified as mediating resistance upon BRAF inhibitor treatment in melanoma [101]. Several mechanisms contributing to BRAF inhibitor-mediated acquired resistance may involve the formation of BRAF splice variants, BRAF gene amplification, and secondary activating mutations such as in RTKs, KRAS, NRAS, or MEK [102,103]. After vemurafenib treatment in BRAFV600E-mutant melanoma, the formation of p61*BRAFV600E* splice variants that can dimerize was found to drive the development of resistance [71]. The bypass activation of other associated signaling pathways can be another mechanism of adaptive resistance. Indeed, activation of the phosphatidylinositol 3-kinase (PI3K)/protein kinase B (AKT) pathway through RTK overexpression, *phosphatase and tensin homolog* (*PTEN*) loss of function, or activating mutations in phosphatidylinositol 3-kinase catalytic subunit (PI3KC) and AKT mediates the development of resistance to BRAF inhibition [104,105]. In *BRAFV600*-mutant NSCLC, resistance can occur after treatment with either BRAF inhibitors as single agents or upon dual BRAF and MEK inhibition by activating mutations in *KRAS* or *NRAS* [74,106,107]. These mutations include *KRASG12D* upon dabrafenib treatment and *NRASQ61K* upon dabrafenib and trametinib treatment. The clinical trial MATCH-R “Matching Resistance” identified that *MEKK57N*, *KRASQ61R*, and *NRASQ61K* mutations and a frameshift *PTEN* mutation in patients progressed to dual BRAF and MEK inhibition using dabrafenib and trametinib, respectively [108]. Another study reported, upon combined BRAF and MEK inhibition, transcriptional upregulation of fibroblast growth factor-1 (FGF-1), which resulted in autocrine fibroblast receptor (FGFR) activation, thus inducing MAPK pathway reactivation [109]. FGFR overexpression has been reported as a resistance mechanism to trametinib in *KRAS*-mutant NSCLC models [110]. Recently, elevated levels of cyclin-dependent kinase 4 (CDK4), a key cell cycle promoting protein, have been observed in a BRAFV600E-mutant NSCLC patient, following dabrafenib and trametinib co-treatment, and the ectopic expression of CDK4 in patient-derived BRAFV600E-mutant cells conferred partial resistance to dabrafenib [111]. Mutations in *mixed lineage kinase 1* (*MLK1*) and *Ras-related C3 botulinum toxin substrate 1* (*RAC1*) can lead to resistance mechanisms independent of RAF activation. Similarly, mutations in *mitogen-activated protein kinase kinases 1* and *2* (*MAP2K1* and *MAP2K2*), the genes that encode for MEK1 and MEK2, respectively, have been observed in both *BRAFV600*-mutant and *BRAF non-V600*-mutant tumors, with *MEK*-mutants exhibiting varying degrees of dependence on RAF for activation [112,113]. Certain *MEK* mutations, like *MEK1 E102_I103del*, confer resistance to allosteric MEK inhibitors but remain sensitive to ATP-competitive MEK inhibitors [114,115]. Downstream alterations, such as amplification of *CCND1*, which encodes for cyclin-D1 and loss of function of *CDKN2A*, which encodes for both p16INK4A and p14ARF, also contribute to resistance to RAF inhibitors [94,116,117,118]. Combinations of MEK or RAF inhibitors with CDK4/6 inhibitors have shown promise in preclinical and early clinical studies and are currently under further clinical investigation [Table 1 and Table 2] [94,118]. Thus, molecular profiling of co-occurring mutations is crucial for detecting possible acquired resistance mechanisms and guiding targeted therapy selection.

## 5. BRAF and MEK Inhibitor Toxicities

The therapeutic benefits of RAF and MEK inhibitors in the management of NSCLC come with a spectrum of toxicities and adverse events that necessitate careful consideration. Common toxicities associated with RAF inhibitors include cutaneous reactions such as rash and photosensitivity, often requiring dose adjustments or temporary treatment interruptions, while hypertension, pyrexia, and increased liver enzymes are the most common adverse events [119,120,121]. Additionally, MEK inhibitors are known to induce gastrointestinal toxicities, including diarrhea, nausea, and vomiting, or may cause interstitial lung disease (Table 3) [120]. Although these side effects are generally manageable, they can impact patients’ quality of life and treatment compliance. Collaborative efforts are essential to comprehensively understand the toxicological profiles and devise strategies for mitigating adverse events to optimize the therapeutic potential of RAF and MEK inhibitors in NSCLC treatment. Ongoing research and clinical trials aim to refine the use of these agents, offering a balance between efficacy and tolerability to improve patient outcomes.

## 6. Discussion

The successful implementation of immune checkpoint inhibitors (ICIs) in the management of melanoma and other cancers has directed efforts to targeted therapy/immunotherapy approaches. Thus far, these attempts have been largely fruitless due to overlapping toxicities that led to early treatment termination [122,123]. Despite that, a triple combination of atezolizumab, an anti-PD-L1 monoclonal antibody, with vemurafenib plus cobimetinib was granted FDA approval in 2020 as a first-line therapy option for patients with advanced BRAFV600E-mutant melanoma [124]. Several ongoing clinical trials are evaluating the combination of BRAF and/or MEK inhibitors together with immune checkpoint inhibitors (ICIs) (Table 1 and Table 2). This strategy is supported by preclinical evidence indicating a synergistic activity of MEK inhibition with immune checkpoint blockade. The biological rationale of this approach relies on the observed immunomodulatory effects of MAPK inhibition in the tumor microenvironment, such as the transcriptional decrease in PD-L1 expression, upregulation of MHC-I expression in tumor cells, and increased pro-inflammatory cytokine production [125,126]. In addition, the RAF-MEK clamp inhibitor avutometinib has been shown, in preclinical models, to induce the antitumor activity of ICIs [127]. Thus, a clinical trial is testing the efficacy and tolerability of the triple combination of the MEK inhibitor selumetinib, in continuous or intermittent dosing schedules, plus durvalumab, an anti-PD-L1, and tremelimumab, an anti-CTLA4, as antibodies in advanced or metastatic NSCLC patients (NCT03581487, Table 2) [128]. Furthermore, this approach is supported by the observed PD-L1 expression in BRAF-mutants and by the enhanced T cell-mediated immunity from BRAF and MEK inhibitors. There is evidence suggesting that BRAF and MEK inhibition induces CD4 and CD8 T lymphocytes, boosting their cytotoxicity against cancer cells, which is evident by elevated granzyme B and perforin levels, and an elevated expression of cytotoxic T-lymphocyte-associated protein-4 (CTLA-4) [111,129,130,131,132,133]. Targeting of CTLA-4 together with MEK, using trametinib or selumetinib, resulted in increased survival in KRAS-mutant lung cancer mouse models [134,135]. In an advanced *BRAFV600E*-mutant NSCLC case study, the combination of atezolizumab and platinum-based chemotherapy led to a prolonged response [135]. The combination of atezolizumab and cobimetinib has been further investigated in a trial with various solid tumors, in which the NSCLC cohort had an ORR of 18% and a median OS of 13.2 months, with a 12-month OS of 57% [136]. An ongoing multicohort clinical study is testing, among other treatments, the triple combination of atezolizumab plus vemurafenib and cobimetinib in *BRAFV600E*-mutant NSCLC patients. The early data of the triple combo suggest further investigation (NCT03178552, Table 2).

While new combinatorial strategies may help in addressing the development of therapeutic resistance, the advancement in the medicinal chemistry field provides many new small molecule inhibitors and targeting agents, like Proteolysis Targeted Chimeras (PROTACs), with unique and improved biochemical properties that may provide better outcomes. For instance, the use of the next-generation type 1.5 (αC-helix OUT) RAF inhibitor PLX8394, which abolishes the paradoxical ERK activation “paradox-breaker”, prevented the BRAF inhibitor monotherapy-mediated drug resistance in BRAFV600E-mutant NSCLC [137]. Moreover, the same compound had additional preclinical activity in certain *BRAF non-V600* NSCLC models [137], indicating that a RAF inhibitor with distinct biochemical characteristics can provide additional benefits. HLX208 is another, more recent, novel RAF monomer-selective inhibitor that has shown promising efficacy and a well-tolerated safety profile in *BRAFV600E*-mutant adult patients with Langerhans cell histiocytosis (LCH) and/or Erdheim–Chester disease (ECD) [138,139]. Currently, HLX208 is being evaluated in a clinical trial of solid tumors in combination with trametinib (NCT04965220, Table 1).

In a different strategy, the concept of implementing a prolonged on–off schedule or introducing “drug holidays” has been suggested as a strategy to favor the expansion of sensitive clones to targeted therapy over resistant ones, thereby maintaining tumor responsiveness [140,141,142]. A similar approach has been proposed in a recent case of successful re-challenge therapy with combined BRAF and MEK inhibition following a series of subsequent chemotherapy treatments. The response mechanism may involve the establishment of a “drug-free” environment, leading to a decrease in the number of heterogeneous tumor cells previously exposed to BRAF and MEK inhibitors, ultimately resulting in a successful re-challenge for certain patients [143]. Downstream inhibition of the terminal pathway kinase, ERK, represents a promising approach to suppress any upstream pathway activation or reactivation. Currently, there are several ERK inhibitors under preclinical and clinical evaluation for MAPK-driven cancers [144,145].

Autophagy, a catabolic cellular process that replenishes nutrients and restores damaged organelles, has been identified as s resistance mechanism to combined BRAF and MEK inhibition in melanoma [146]. Targeting autophagy together with BRAF and MEK inhibitors has provided clinical benefit for advanced BRAFV600E-mutant melanoma [147,148]. Unc-51-like kinase (ULK) kinase integrates inputs from nutrient and stress sensors to initiate autophagy [146]. DCC-3116 is a potent and selective inhibitor of ULK that has exhibited preclinical evidence and is now under clinical evaluation in combination with trametinib, binimetinib, or sotorasib, a KRASG12C inhibitor, for MAPK-driven cancer (NC04892017, Table 2) [149]. Alternatively, co-targeting pyroptosis, an alternative type of non-apoptotic, inflammation-related programmed cell death, with MAPK pathway inhibitors seems an attractive approach [150,151]. This is supported by the absence of pyroptosis-related markers in BRAF and MEK-combined inhibition-resistant *BRAF*-mutant cancer models, which was associated with reduced T cell antitumor immunity and, importantly, was sensitive to pyroptosis-inducing chemotherapy [152].

SHP2 phosphatase has been an attractive antitumor target, mainly because of its key role as a signal transduction node downstream of multiple RTKs and, subsequently, as a positive regulator of RAS and MAPK signaling [153,154,155]. More specifically, SHP2 inhibitors have shown efficacy against tumors harboring class III *BRAF* mutations, certain *KRAS* mutations, such as *KRASG12C*, *EGFR* mutations, or with *NF1* loss [156,157,158]. However, resistance to single-agent SHP2 inhibition or combination with MEK inhibition has been observed in cases of *KRASG13D* and *KRASQ61* mutations, and some BRAFV600E models. This resistance was attributed to FGFR-mediated, SHP2-independent feedback reactivation of RAS signaling pathways [156]. In a first-in-human trial of various tumor models, including *BRAFV600E*-mutant colorectal cancer and *KRASG12D*-mutant ovarian cancer, after disease progression on monotherapy with PF-07284892, an allosteric SHP2 inhibitor, a combination of encorafenib with binimetinib was administered in addition to PF-07284892. The triple combination therapy led to tumor response and to prolongation of the overall clinical benefit [159,160]. This clinical study provides a rationale for the utility of RAF and MEK inhibitors in combination with SHP2 inhibitors in overcoming resistance to targeted therapies, presenting a valuable model for testing novel drug combinations early in clinical development.

### 6.1. Future Directions—Next-Generation RAF Inhibitors

There is preclinical evidence that next-generation αC-helix IN RAF inhibitors, and more specifically, the subgroup of RAF dimer selective inhibitors, may provide benefits in targeting BRAF *non*-*V600* mutants (class II and III BRAF mutations) in NSCLC [43]. However, in the absence of comprehensive studies, the current guidelines for clinicians suggest that *BRAF non-V600E* mutants should be managed as non-mutationally-driven NSCLC [161]. Naporafenib, a RAF dimer selective inhibitor, in combination with the MEK inhibitor trametinib or in combination with the ERK inhibitor LTT462, is being explored in a clinical trial for its efficacy in advanced metastatic *KRAS-* or *BRAF*-mutant NSCLC or in *NRAS*-mutant melanoma (NCT02974725, Table 1). Preliminary results from the *NRAS*-mutant cohort demonstrated a promising ORR of 47% for the first 15 enrolled patients [162]. Lifirafenib (BGB-283), another RAF inhibitor of the same category (αC-IN, equipotent for RAF monomers and dimer), has shown antitumor activity in *KRAS*-mutant NSCLC [70]. Currently, lifirafenib in combination with the MEK inhibitor mirdametinib is being evaluated in a phase I clinical trial (NCT03905148, Table 1) with preliminary findings indicating a favorable safety profile and showing evidence of antitumor activity, including in NSCLC patients harboring *KRAS*, *NRAS*, and *BRAF* mutations. [147]. These results set the base for further exploration of these next-generation RAF inhibitors beyond *BRAF*-mutant NSCLC. In a phase Ib trial, belvarafenib, another dimer selective RAF inhibitor, in combination with the MEK inhibitor cobimetinib, demonstrated a partial response in two *BRAF non-V600*-mutant NSCLC patients tested [163]. An ongoing phase I multicenter study is testing belvarafenib, in combination with cobimetinib or with the anti-EGFR monoclonal antibody cetuximab, in locally advanced or metastatic solid tumors (NCT03284502, Table 1).

### 6.2. Biomarkers—Predictors of Response to RAF and MEK Inhibitors

The National Comprehensive Cancer Network (NCCN) NSCLC panel currently recommends *BRAF* mutation testing for of all patients with metastatic non-squamous NSCLC and suggests the combination targeted therapy of dabrafenib plus trametinib as a preferred first-line therapy for BRAFV600E-mutant NSCLC patients [164]. Drawing insights from recent studies, predictive biomarkers offer valuable insights into treatment response and outcomes. The first study highlights the potential of specific biomarkers, including p27 accumulation, cyclin D1 downregulation, PARP cleavage, and increased phospho-4E-BP1, in predicting cellular responses to trametinib treatment, indicating a spectrum of outcomes ranging from apoptosis to autophagy, providing a framework for understanding treatment efficacy [165]. Moreover, insights from a more recent study underscore the limitations of combined RAF and MEK inhibition in NSCLC patients with activated *BRAF non-V600* mutations. Despite well-tolerated treatment regimens, incomplete ERK pathway inhibition was observed in on-treatment biopsies, suggesting a need for more robust predictive biomarkers to identify patients likely to benefit from this therapeutic approach [166]. Moreover, the recent association of phenotypic and spatial characteristics of cancer-associated fibroblasts (CAFs) in the tumor microenvironment of NSCLC, with patient outcome [167], is worth exploring further in the context of RAF and MEK inhibitor targeted therapies in *BRAF*-mutant NSCLC. Overall, these findings underscore the importance of a comprehensive molecular profiling and personalized medicine in NSCLC management, emphasizing the critical role of predictive biomarkers in optimizing treatment strategies and improving patient outcomes.

## 7. Conclusions—Future Perspectives

The ongoing advancements in the development of the next generation of RAF and MEK inhibitors, characterized by distinct biochemical properties, present promising avenues for the treatment of lung cancer. These novel inhibitors are currently undergoing rigorous preclinical and clinical evaluations. Their exploration offers a potential solution to the challenges posed by adaptive resistance and the applicability of existing inhibitors to specific mutational profiles, such as *BRAFV600E*-mutant NSCLC patients. Moreover, the evolving landscape of precision medicine and targeted therapies continues to fuel optimism for overcoming these challenges. In conclusion, while the approvals of the dabrafenib–trametinib and encorafenib–binimetinib combinations represent significant strides in the treatment of *BRAFV600E*-mutated NSCLC, the field is actively working towards more inclusive and effective therapeutic strategies. Improved targeting agents moving into the clinic and rational combinatorial strategies, targeting selectively key components “inside” and “outside” the MAPK pathway, have become more beneficial. The very recently granted fast track designation for the combination of the RAF−MEK clamp inhibitor avutometinib plus the KRASG12C selective inhibitor sotorasib for the treatment of *KRASG12C*-mutant NSCLC patients underscores the continuing potential of RAF and MEK inhibition against RAS/MAPK-driven malignancies. The ongoing research on next-generation inhibitors and the expansion of targetable mutations hold promise for a more comprehensive and adaptable approach to address drug resistance and widen the therapeutic impact of RAF and MEK inhibition in the diverse landscape of lung cancer.

## Figures and Tables

**Table 1:** RAF and MEK inhibitors as monotherapies or in combination with other compounds under clinical evaluation in lung cancer. RAF, rapidly accelerated fibrosarcoma; BRAF, v-RAF murine sarcoma viral oncogene homolog B; MEK, mitogen-activated protein-kinase kinase; ERK, extracellular signal-regulated kinase; KRAS, Kirsten rat sarcoma viral oncogene homolog; mTOR, mechanistic target of rapamycin; CDK4/6, cyclin-dependent kinase 4/6; PD-1, programmed cell death protein 1; PD-L1, programmed death-ligand 1; VEGFR, vascular endothelial growth factor receptor; FGFR, fibroblast growth factor receptor; PDGFR, platelet-derived growth factor receptor; KIT, tyrosine-protein kinase; EGFR, epidermal growth factor receptor; RET, rearranged during transfection. Retrieved from clinicaltrials.gov (assessed on 27 February 2024).

Drug(s)	Target(s)	Second Drug(s)	Second Target(s)	Trial Phase	Clinical Trial Identifier
BGB-3245	RAF	-	-	I	NCT04249843
BDTX-4933	RAF	-	-	I	NCT05786924
Exarafenib	RAF	-	-	I	NCT04913285
FORE8394	RAF	-	-	I/II	NCT02428712
ABM-1310	RAF	+/− Cobimetinib	MEK	I	NCT04190628
Belvarafenib	RAF	Cobimetinib Cetuximab *	MEK EGFR	I	NCT03284502
BGB-3245	RAF	Mirdametinib	MEK	I/II	NCT05580770
Dabrafenib	ΒRAFV600E	Trametinib	MEK	IV	NCT03340506
Encorafenib	ΒRAFV600E	Binimetinib	MEK	II	NCT03915951
Encorafenib	ΒRAFV600E	Binimetinib	MEK	II	NCT05195632
Encorafenib	ΒRAFV600E	Binimetinib	MEK	II	NCT04526782
Encorafenib	ΒRAFV600E	Binimetinib	MEK	II	NCT03839342
HLX208	ΒRAFV600E	Trametinib	MEK	I	NCT04965220
Lifirafenib	RAF	Mirdametinib	MEK	I	NCT03905148
Naporafenib	RAF	Trametinib	MEK	I	NCT05907304
Naporafenib	RAF	Trametinib LTT462 Ribociclib	MEK ERK CDK4/6	I	NCT02974725
PF-07799933	RAF	Binimetinib Cetuximab *	MEK EGFR	I	NCT05355701
Tinlorafenib	RAF	Binimetinib	MEK	I	NCT04543188
Tovorafenib	RAF	+/− Pimasertib	MEK	I/II	NCT04985604
Vemurafenib	ΒRAFV600E	Cobimetinib	MEK	II/III	NCT05768178
XP-102	RAF	+/− Trametinib	MEK	I/II	NCT05275374
ABM-168	MEK	-	-	I	NCT05831995
IMM-6-415	MEK	-	-	I/II	NCT06208124
IMM-1-104	MEK	(Chemotherapy)	-	I/II	NCT05585320
Avutometinib	MEK/RAF	Sotorasib	KRASG12C	I/II	NCT05074810
Avutometinib	MEK/RAF	+/− Everolimus	mTOR	I	NCT02407509
Binimetinib	MEK	Palbociclib	CDK4/6	I/II	NCT03170206
Binimetinib	MEK	Pembrolizumab	PD-1	I	NCT03991819
Cobimetinib	MEK	Atezolizumab	PD-L1	II	NCT03600701
Mirdametinib	MEK	Palbociclib	CDK4/6	I/II	NCT02022982
PF-07799544	MEK	Tinlorafenib PF-07799933 Encorafenib	RAF	I	NCT05538130
Selumetinib	MEK	Docetaxel ^†^	Microtubule depolymerization	III	NCT01933932
Selumetinib	MEK	Osimertinib	EGFRL858R/T790M	II	NCT03392246
Trametinib	MEK	Anlotinib	VEGFR, FGFR, PDGFR, KIT	I	NCT04967079
Trametinib	MEK	EGF816	EGFR	I	NCT03516214
Trametinib	MEK	Everolimus	mTOR	II	NCT04803318
		Lenvatinib	VEGFR, FGFR, PDGFR, KIT, RET		
Trametinib	MEK	Pembrolizumab	PD-1	I	NCT03299088
Trametinib	MEK	Pembrolizumab	PD-1	I/II	NCT03225664
Trametinib	MEK	Docetaxel ^†^	Microtubule depolymerization	II	NCT02642042

* monoclonal antibody, ^†^ chemotherapy medication.

**Table 2:** RAF and MEK inhibitors (highlighted) as secondary treatment or as sub-study treatment in multidrug testing trials under clinical evaluation in lung cancer. SOS1, son of sevenless homolog; MEK, mitogen-activated protein-kinase kinase; KRAS, Kirsten rat sarcoma viral oncogene homologue; PD-1, programmed cell death protein 1; TIGIT, T cell immunoreceptor with Ig and ITIM domains; BRAF, v-RAF murine sarcoma viral oncogene homolog B; VEGFR, vascular endothelial growth factor receptor; VEGF-A, vascular endothelial growth factor-A; PDGFRβ, platelet-derived growth factor receptor β; SHP2, Src homology 2 domain-containing phosphatase 2; ROS1, c-ros oncogene-1; MET, hepatocyte growth factor receptor; ALK, anaplastic lymphoma kinase; EGFR, epidermal growth factor receptor; CDK4/6, cyclin-dependent kinase 4/6; PD-L1, programmed death-ligand 1; CTLA-4, cytotoxic T-lymphocyte-associated protein 4; HER2, human epidermal growth factor receptor 2; RET, rearranged during transfection; WEE-1, Wee1-like protein kinase; AKT, protein kinase B; SRC, proto-oncogene tyrosine-protein kinase; BCR-ABL, breakpoint cluster region protein–tyrosine-protein kinase Abl1; FAK, focal adhesion kinase; FGFR1-4, fibroblast growth factor receptor 1-4; TRKA, tropomyosin receptor kinase A; PI3Kα,β,γ,δ, phosphatidylinositol 3-kinase-α,β,γ,δ; LAG-3, lymphocyte-activation gene 3; mTOR, mechanistic target of rapamycin; PI3KCA, phosphatidylinositol 3-kinase catalytic subunit alpha; SMO, smoothened; P-gp, permeability glycoprotein; ABCG2, ATP-binding cassette super-family G member; ATR, ataxia telangiectasia and Rad3-related protein. Retrieved from clinicaltrials.gov (assessed on 27 February 2024).

Drug(s)	Target(s)	Second Drug(s)	Second Target(s)	Trial Phase	Clinical Trial Identifier
BI-1701963	SOS1	**+/− Trametinib**	**MEK**	I	NCT04111458
DCC-3116	ULK1/2	**Trametinib** **Binimetinib** Sotorasib	**MEK** **MEK** KRASG12C	I/II	NC04892017
Sasanlimab	PD-1	**Encorafenib/binimetinib** Axitinib /SEA-TIGIT *	**ΒRAFV600E** **MEK** VEGFR1-3, PDGFRβ TIGIT	I/II	NCT04585815
PF-07284892	SHP2	**Encorafenib** **Binimetinib** Lorlatinib Cetuximab *	**ΒRAFV600E** **MEK** ROS1, ALK EGFR	I	NCT04800822
Opnurasib	KRASG12C	**Trametinib** Ribociclib Cetuximab *	**MEK** CDK4/6 EGFR	I/II	NCT05358249
Durvalumab * Tremelimumab *	PD-L1 CTLA-4	**Selumetinib**	**MEK**	I/II	NCT03581487
Alectinib	ALK	**Cobimetinib**	**MEK**	I/II	NCT03202940
Caboplatin ^†^/pemetrexed ^†^/pembrolizumab *	PD-1	**Mirdametinib**	**MEK**	I/II	NCT05937906
**Vemurafenib** Alectinib Atezolizumab * Trastuzumab emtansine ^#^	**BRAFV600E** ALK PD-L1 HER2-microtubulin	-	-	II	NCT02314481
Atezolizumab *	PD-L1	**Cobimetinib** **/vemurafenib**	**MEK** **BRAFV600E** VEGF-A	II/III	NCT03178552
Atezolizumab *	PD-L1	Bevacizumab * /pemetrexed ^†^ /carboplatin ^†^			
Atezolizumab * Alectinib Entrectinib	PD-L1 ALK ROS1				
Divarasib	KRASG12C	Docetaxel ^†^	Microtubule depolymerization		
**Vemurafenib**	**BRAFV600E**	**Cobimetinib**	**MEK**	II	NCT04302025
Alectinib Entrectinib Pralsetinib Atezolizumab * Divarasib	ALK ROS1 RET PD-L1 KRASG12C	-	-		
**Dabrafenib**	**BRAFV600E**	**Trametinib**	**MEK**	II	NCT02465060
**Binimetinib** **Trametinib** Ulixertinib Adavosertib Afatinib Capivasertib Copanlisib Crizotinib Dasatinib Defactinib Erdafitinb Fexagratinib Ipatasertib Larotrectinib Osimertinib Palbociclib GSK2636771 Sapanisertib Sunitinib Taselisib Vismodegib	**MEK** **MEK** ERK WEE-1 EGFR, HER2 AKT PI3Kα,β,γ,δ ALK, MET, ROS1 SRC, BCR-ABL FAK FGFR1-4 FGFR1-4 AKT TRKA, B and C EGFRL858R/T790M CDK4/6 PI3Kβ mTOR VEGFR2, PDGFRβ PI3KCA, PI3Kα,β,γ,δ SMO, P-gp, ABCG2	**-**	**-**		
Pertuzumab *	HER2	Trastuzumab *	HER2		
Nivulomab *	PD-1	Relatlimab *	LAG-3		
**Selumetinib**	**MEK**	Docetaxel ^†^	Microtubule depolymerization	II	NCT02664935
Ceralasertib	ATR	Durvalumab *	PD-L1		
Sitravatinib	Axl, MER, VEGFR1-3, KIT, FLT3, DDR1-2, TRKA and B				
Capivasertib Fexagratinib Vistusertib Palbociclib Crizotinib Osimertinib	AKT FGFR1-4 mTOR CDK4/6 ALK, MET, ROS1 EGFRL858R/T790M				

* monoclonal antibody, ^†^ chemotherapy medication, ^#^ antibody–drug conjugate.

**Table 3:** Key clinical trial outcomes for BRAFV600E-mutant NSCLC patients upon treatment with RAF and/or MEK inhibitors and their most frequent adverse events. ORR, objective response rate; TRAEs, treatment-related adverse events.

Drug(s)	Target(s)	Number of Patients	ORR	TRAEs	Reference
Encorafenib + binimetinib	BRAFV600E MEK	98	75%	Nausea, diarrhea, fatigue	[91]
Dabrafenib + trametinib	BRAFV600E MEK	93	68.4%	Pyrexia, nausea, vomiting	[87]
Dabrafenib + trametinib	BRAFV600E MEK	36	64%	Pyrexia, nausea, fatigue, peripheral oedema	[86]
Dabrafenib	BRAFV600E	84	33%	Pyrexia, hyperkeratosis, decreased appetite	[47]
Vemurafenib	BRAFV600E	101	44.9%	Nausea, hyperkeratosis, decreased appetite	[66]
Cobimetinib + atezolizumab *	MEK PD-L1	28	18%	Diarrhea, rash, fatigue	[94]

* monoclonal antibody.

## Data Availability

Not applicable.
